# Vehicle Operation Status Monitoring Based on Distributed Acoustic Sensor

**DOI:** 10.3390/s23218799

**Published:** 2023-10-29

**Authors:** Mengmeng Chen, Haotian Ding, Mingming Liu, Zhigao Zhu, Dongdong Rui, Ye Chen, Fei Xu

**Affiliations:** 1 College of Electronic Engineering, Nanjing Xiaozhuang University, Nanjing 211171, China; chenmm@njxzc.edu.cn; 2 College of Engineering and Applied Sciences, and Collaborative Innovation Center of Advanced Microstructures, Nanjing University, Nanjing 210023, China; dg21340001@smail.nju.edu.cn; 3 Railway Vehicle Department, Nanjing Vocational Institute of Railway Technology, Nanjing 210031, China; lmqfly@163.com; 4 CRRC Nanjing Puzhen Co., Ltd., Nanjing 210031, China; 010700080501@crrcgc.cc (Z.Z.); 010700080818@crrcgc.cc (D.R.); 5 College of Physics, Nanjing University of Aeronautics and Astronautics, Nanjing 211106, China; yechen@nju.edu.cn

**Keywords:** distributed acoustic sensor, distributed optical fiber sensor, vehicle monitoring, field test

## Abstract

To develop implementation research on distributed optical fiber sensing technology, field tests were conducted on municipal roads and railways using a distributed acoustic sensor (DAS). Data were collected by the DAS during a field test for a long time period (more than 20 min), and we conducted short-term (<10 s) and long-term (≥10 s) analyses on these data separately. In the short-term data analysis, the vehicle type, vehicle length, and working status of the vehicle engine or the compressor were identified. In the long-term data analysis, the traffic flow was monitored, and the running distance, acceleration, speed, and braking distance of the vehicle were obtained. The characteristics of the vehicle operation data obtained in these field tests are important in developing the data processing method of DASs, which will help to promote the implementation of DASs.

## 1. Introduction

A distributed acoustic sensor (DAS) is a kind of distributed optical fiber sensing technology that can monitor vibration, strain, and acoustic events using conventional communication optical cables. The phase-sensitive time-domain reflectometer (Φ-OTDR) is a natural technical solution for DASs. Since being proposed by Taylor et al. in 1993, the Φ-OTDR has received widespread attention [1]. The most popular advantage of this technology is that it can quantify the strength of vibration or acoustic events through demodulated phase information. Therefore, one of the most important technologies in the DAS system is how to demodulate the phase. Normally, there are four methods for the demodulation of the phase: orthogonal (IQ) phase demodulation, Hilbert transform phase demodulation, 3×3 coupler-based phase demodulation, and phase-generated carrier (PGC) demodulation.

IQ phase demodulation is mainly used for heterodyne coherent detection structures. The beat frequency signal output by the BPD is multiplied by the two local orthogonal digital signals, and the two obtained results are filtered by a low-pass filter to remove the second harmonic term and noise. The arctangent is then taken to obtain the wrapped phase information [2]. The Hilbert-transform-based phase demodulation method is used to first perform the Hilbert transform on the signal, then divide it by the original signal and take the arctangent, and finally pass it through a low-pass filter to obtain the wrapped phase information [3]. The 3×3-coupler-based phase demodulation method acquires the phase information from the MZI (Mach–Zehnder interferometer) combined with a 3×3 coupler, which introduces a 120° phase difference [4,5,6]. The PGC-based phase demodulation method obtains the phase information by introducing a non-balanced Michelson interferometer (MI) and the PGC modulation/demodulation method [7,8]. The PGC differential crossover (PGC-DCM) algorithm and PGC arctangent method are the two most widely used standard PGC algorithms.

These four phase demodulation methods each have their own advantages and disadvantages, and they need to be selected based on actual situations. The IQ demodulation structure is relatively simple, with a high signal-to-noise ratio (SNR) and sensitivity but can only be used for heterodyne coherent detection. Hilbert transform demodulation can be used for direct detection and homodyne coherent detection, but due to its inability to eliminate low-frequency noise in low-pass filtering, its ability to resist noise is much inferior to that of IQ demodulation. The 3×3 coupler demodulation method is suitable for the direct detection of the optical path and is less affected by polarization noise but requires high device consistency. The PGC demodulation method can also be applied to the direct detection of optical paths, but it requires the high stability of the modulator working station. 

In general, DAS technology has been developed to a relatively mature stage, but there are still relatively few data analyses for specific engineering applications. In the past several decades, communication operators have established large-scale fiber optic infrastructure. However, the fiber optic infrastructure only provides data transmission functions and has reserved a large amount of affluent optical fiber so far. The development of distributed optical fiber sensor networks using the abundant fiber of existing fiber optic communication networks has become a new field explored by scientists [9,10,11]. With the increasing development of social intelligence, the demand for actionable information for the effective management of transportation networks in smart transportation continues to grow. This new aim of utilizing fiber optic infrastructure to build distributed fiber optic sensing networks has significant value in smart transportation applications. DASs are considered for use in this field because they provide some unique features compared to other sensing technologies, such as circular detectors, magnetic sensors, radar sensors, microwave detectors, cameras, etc. This is related to the distributed monitoring features [12,13] of DASs.

Vehicle status monitoring is an important goal of an intelligent transportation system. Covering and analyzing several kilometers of a road network with cameras are extremely challenging and costly, and there are inevitably limitations such as blind areas and poor performance in bad weather conditions [14]. The use of DASs based on optical fiber can collect uninterrupted data along the length of the road, which overcomes the shortcomings of other sensors. If the DAS system connects to an optical fiber cable, each point along the fiber becomes a sensing unit, achieving continuous detection along the length of the road. The DAS system can continuously monitor fluctuation signals caused by external vibrations, and these are called acoustic events [15,16,17]. DAS data can be analyzed, which helps to locate acoustic events along optical fibers and classify them (such as cars, trucks, etc.). DASs are a relatively new technology and are expected to overcome blind spots and to reduce deployment costs of equipment and data analyses [18,19,20,21]. More importantly, DASs are not affected by adverse weather or photometric conditions, and they do not capture personal data, such as faces and clothing. 

In this paper, a self-made DAS unit based on the heterodyne detection technical scheme and Hilbert’s phase demodulation method is used for a field test to achieve vehicle status monitoring on railways and municipal roads. The DAS unit can monitor the traffic flow and obtain the running status of the vehicles. The optical data characteristics of different types of vehicles are analyzed, and the running state of the vehicles is also analyzed. The experimental results show that the DAS can obtain good results in vehicle status monitoring and speed estimation. The data characteristics obtained in this field test have important significance for promoting sensor networks based on DASs.

## 2. Experimental Setup and Data Processing 

### 2.1. Experimental Setup

**Figure 1:**
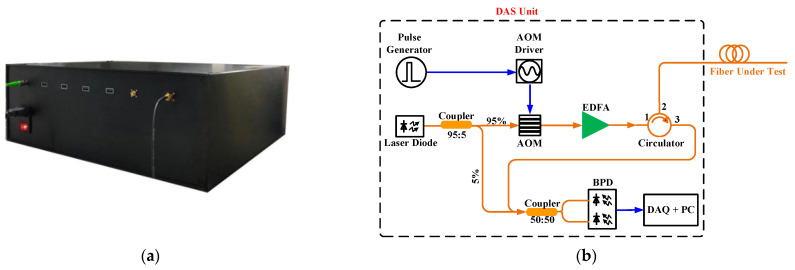
Principle block diagram of the DAS: (**a**) demo picture of the DAS unit and (**b**) technical scheme of the DAS unit.

The DAS unit used in this field test is a self-made unit (refer to Figure 1a); the technical scheme inside the unit is given in Figure 1b. In this unit, a laser with a wavelength of 1550 nm and a power of 20 mW is used as the source, and the light is divided into two parts through a 95:5 coupler. The 95% section is modulated by an AOM (insertion loss = 3.4 dB; extinction ratio = 50 dB) to generate a 30 ns optical pulse which corresponds to a spatial resolution of 3 m with a repetition rate of 20 kHz. Each optical pulse is amplified by an erbium-doped fiber amplifier (EDFA). The amplified pulses are transmitted to the sensing fiber through the first and second ports of the optical circulator, and the second and third ports of the optical circulator are used to collect the backscattered Rayleigh light from the sensing fiber. Then, the backscattered light and the 5% section from the 1550 nm laser are simultaneously injected into the 50:50 coupler, and the outputs of the 50:50 coupler are received by a balanced photodetector (BPD, 30 kV/A transimpedance; 200 MHz bandwidth). Finally, a data acquisition card (DAQ, bandwidth 100 MHz; sampling rate 250 MSa/s) is used to collect the electrical signals’ output by the BPD, and the data are analyzed by a personal computer (PC). The phase demodulation method is based on the method used in Reference [3].

### 2.2. Field Test Setup

**Figure 2:**
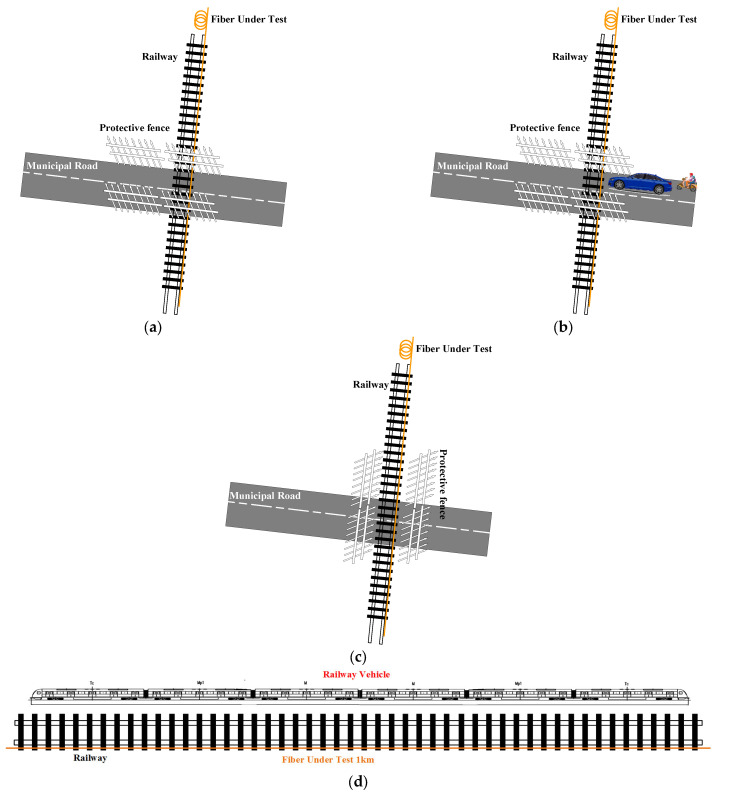
Field test scenes: (**a**,**b**) are the scene for monitoring on the municipal road (the optical fiber cable is perpendicular to the direction of the monitored traffic flow); and (**c**,**d**) are the scene for monitoring on the railway (the optical fiber cable is parallel to the direction of the monitored traffic flow).

The field test was carried out in collaboration with CRRC Nanjing Puzhen Co., Ltd., Nanjing, China. The experiment scene is given in Figure 2. The test field was conducted on their vehicle test track, and Figure 2a is the schematic diagram of the field. The trial operation track intersects with a municipal road in a cross shape, and a 1 km G652 single-mode fiber (SMF) cable was laid on the siding of the railway; the orange line in Figure 2a is the fiber cable. There is a fence on the experimental field to maintain traffic safety. When there is no trial operation of railway vehicles, the fence is parallel with the municipal road, and the municipal road is free for passing through, just as shown in Figure 2b. When there are railway vehicles undergoing trial operation (refer to Figure 2d), the fence is parallel with the railway (refer to Figure 2c) and other motor vehicles are prohibited from passing.

### 2.3. Data Processing

**Figure 3:**
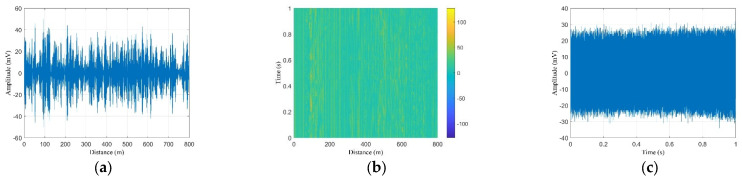
Raw data obtained from the DAS: (**a**) raw data of a single pulse, (**b**) raw data of 20 k pulses, and (**c**) raw data of a single position along fiber (location 162 m).

The raw data acquired by the DAS are shown in Figure 3. First, the system collects time-domain traces with a speed of 20 k traces per second. The time-domain data of the OTDR trace can be converted to distance using the formula z = c*N*∆t/(2n), where z represents the distance, c is the speed of light in vacuum, n is the effective refractive index of the fiber mode, N is the point number along the trace, and ∆t is the sampling interval. Figure 3a shows one typical time-domain trace which lasts 800 m. The sequence of 20 k traces was redrawn to provide a 2D plot matrix of the backscattered traces and allows for the output at a particular position to be determined as a function of time (Figure 3b) [18]. Figure 3c is the time-domain data that last for 1 s of a fixed point (162 m) extracted from Figure 3b, which corresponds to the y-axis direction in Figure 3b.

**Figure 4:**
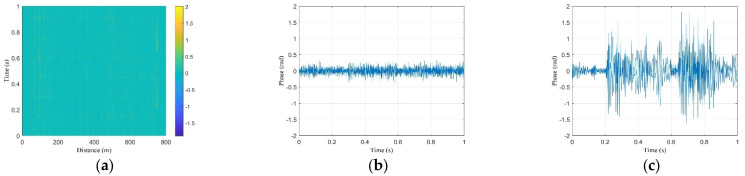
Processed data: (**a**) phase data after demodulation, (**b**) phase noise base (<0.35 rad, (location 240 m)), (**c**) and phase information of a bicycle passing by (location 749 m).

Figure 4a shows the phase data achieved after filtering and phase demodulation of the data in Figure 3b; the phase information reflects whether there is vibration or acoustic event along the fiber. For example, there is no vibration at 240 m (Figure 4b) and there is a vibration at 749 m (Figure 4c). Based on the data in Figure 4b, the noise base of the obtained data after phase demodulation is less than 0.4 rad. During the data feature extraction process, the phases greater than 0.5 radians are treated as vibration events.

Due to the two types of roads in the field test scene (refer to Figure 2a), corresponding to the two directions of the fiber cable, the municipal road is perpendicular to the fiber cable, and the railway is parallel to the fiber cable. The data from the two directions were both analyzed. For the municipal road, the data located from 700 m to 800 m of the fiber cable were analyzed, which precisely correspond to the distribution of fiber cables on the municipal road. For the railway, all the data along the fiber cable were analyzed, ranging from 0 m to 1000 m. For each type of data mentioned above, both can be divided into two categories: short-term analysis and long-term analysis according to the different durations of the selected data. 

**Figure 5:**
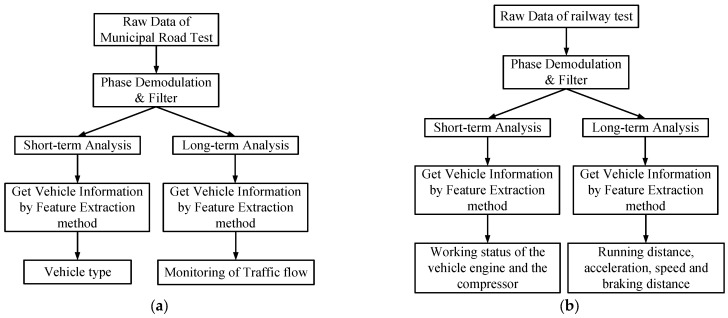
DAS data processing flow chart: (**a**) for the municipal road test and (**b**) for the railway test.

The data processing flowcharts are given in Figure 5. The raw data acquired by the DAS were subjected to phase demodulation [3] and filtering [19,20] before proceeding to the next step of processing, which was divided into long-term analysis and short-term analysis. Both long-term and short-term analyses can obtain vehicle information through feature extraction methods [18], which are described in detail in Section 3. For the municipal road test, the vehicle type can be identified through short-term analysis, and the traffic flow can be monitored through long-term analysis (refer to Figure 5a). For the railway test, the working status of the vehicle engine and the compressor can be monitored through short-term analysis, and parameters such as running distance, acceleration, speed, and braking distance can be obtained through long-term analysis (refer to Figure 5b).

## 3. Vehicle Monitoring

### 3.1. Municipal Road Test

**Figure 6:**
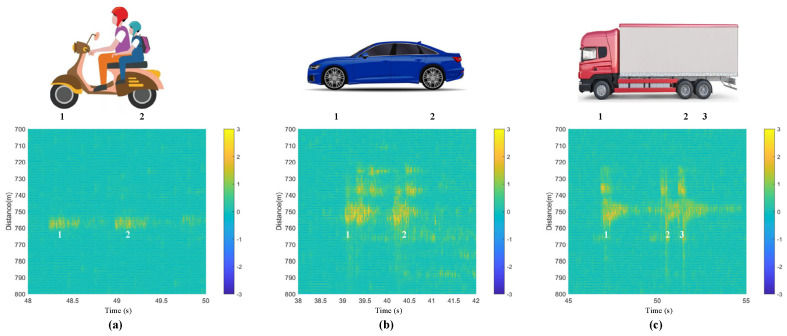

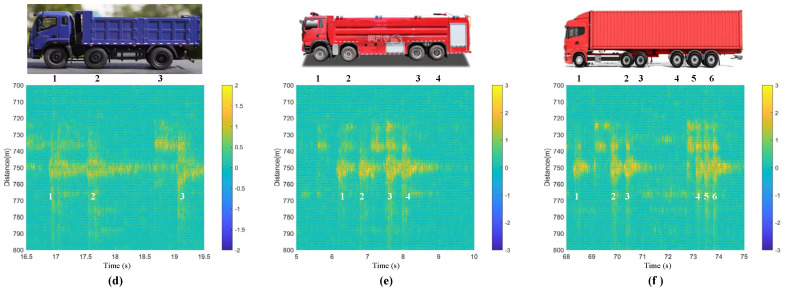
Response characteristics of different types of vehicles: (**a**) a motorcycle, (**b**) a car, (**c**) a container car, (**d**) a three−axle truck, (**e**) a four−axle truck, and (**f**) a six−axle truck.

**Figure 7:**
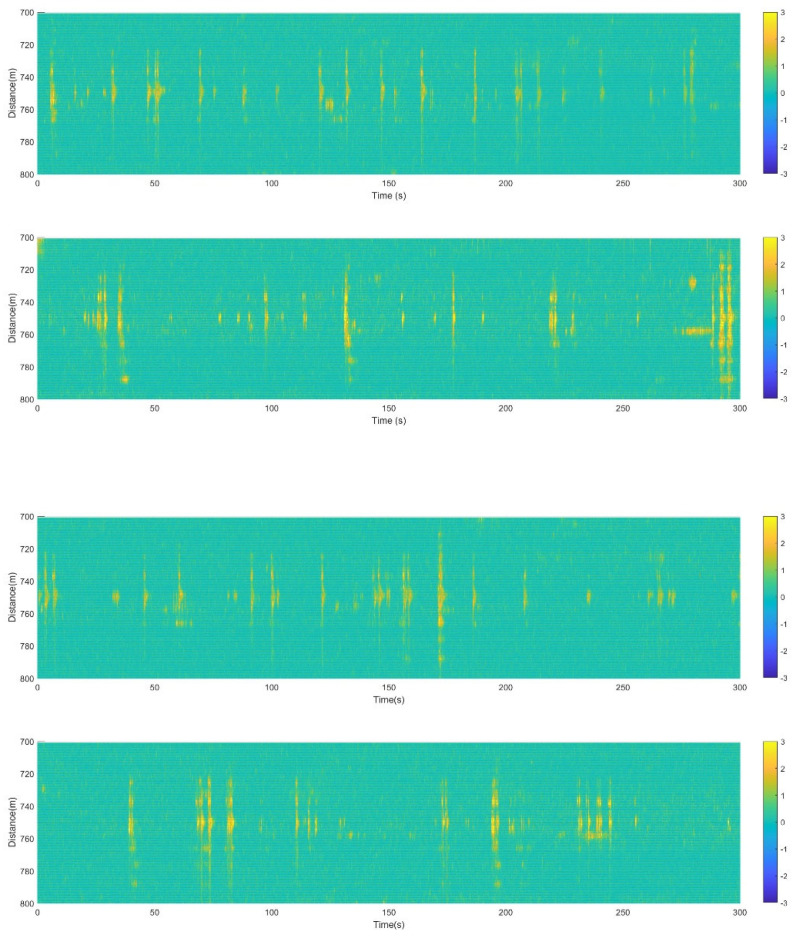
Traffic flow monitoring; each figure lasts 5 min.

**Figure 8:**
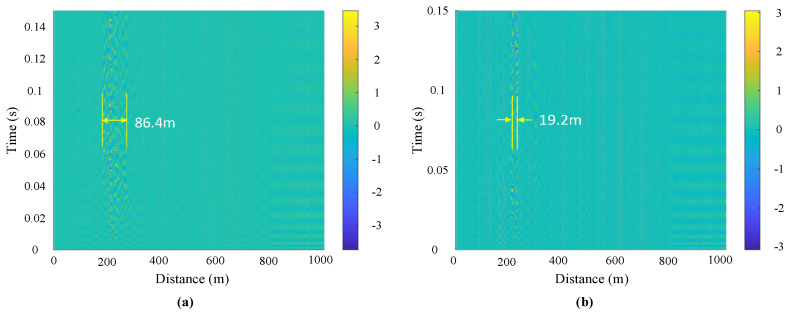
Vehicle status monitoring: (**a**) engine start status and (**b**) compressor working status.

**Figure 9:**
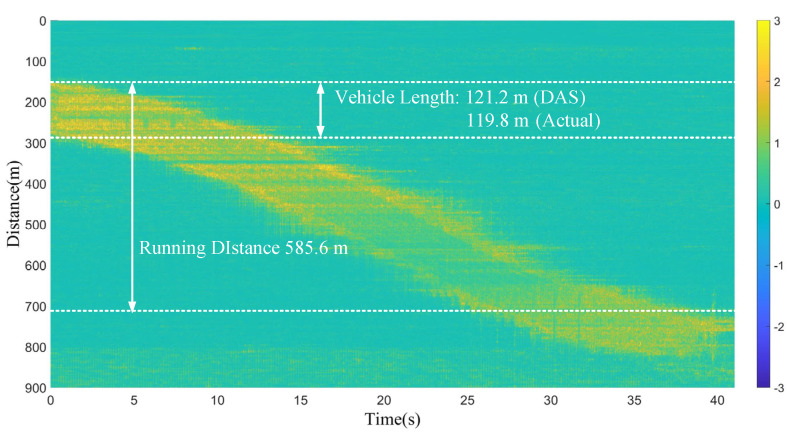
Vehicle length and running distance measured by the DAS.

**Figure 10:**
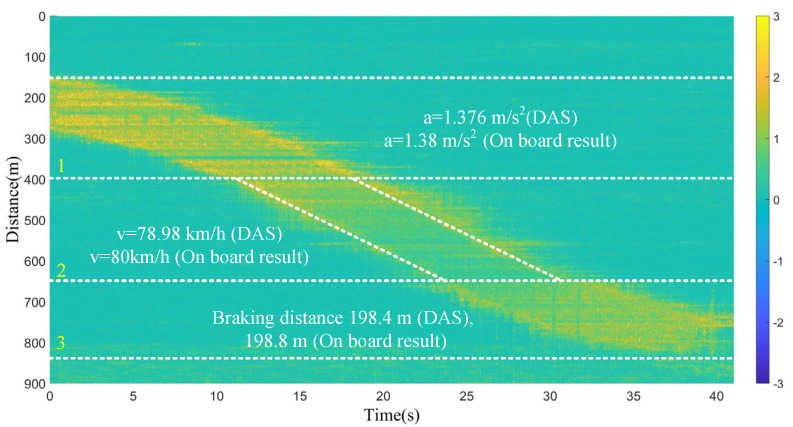
Speed, acceleration, and braking distance measured by the DAS and the on-board system of the railway vehicle.

**Figure 11:**
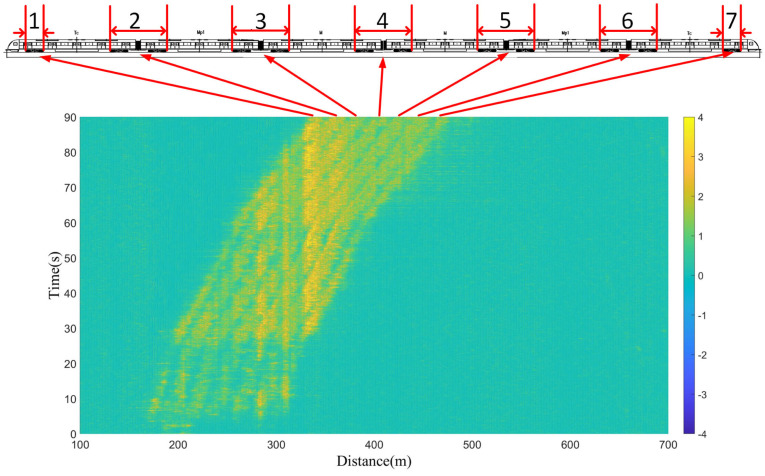
Results of one−to−one correspondence with the axles of the measured vehicle.

There are fences on both sides of the road, and the optical cable crosses the road perpendicularly, with vehicles passing over the cable (refer to Figure 2b). The monitored results are given as follows. For the processed data distribution in Figure 6, Figure 7, Figure 8, Figure 9, Figure 10 and Figure 11, the yellow data in the figures indicate the positive phase, representing the vibration of the vehicle. The characteristics of the vehicles are extracted based on the phase-demodulated data. The specific characteristics of the different types of vehicles passing through the optical cables are different. The six figures in Figure 6 represent six different types of vehicles, with the top of each figure showing the vehicle type and the data on the bottom corresponding to the data features of the above vehicle. When each wheel passes through the optical cable, there is a response characteristic spectral line similar to that in Figure 4c, and the number marked in the figure corresponds to the order of the axles passing through. The number of axles for these six types of vehicles is different, and the number of characteristic spectral lines in the corresponding figure is also different. 

Figure 7 shows the results of the continuous monitoring of the traffic flow for 20 min. For a clearer display of the vehicle information, each figure in Figure 7 represents the data of 5 of the 20 min. As the municipal road is located in a relatively remote suburban area, there are more freight trucks and fewer cars and motorcycles passing by. In the 20 min monitored data, there are 43 trucks, 24 cars, and 12 motorcycles.

### 3.2. Railway Test

In this test, a newly manufactured railway vehicle was used as the measurement sample for the field test. The train consists of six carriages, including four motor carriages and two trailer carriages (refer to Figure 2d). When the railway vehicle is running on the railway, the interaction between the wheels and the railway generates soundwaves which are picked up by the optical fiber cable and induced phase change of the sensing probe light returning to the DAS unit. By analyzing the distribution of the sound waves in time and space, parameters such as the train length, running distance, acceleration, speed, and braking distance can be calculated.

The middle four carriages of this vehicle have engines, and the first and last two carriages do not have engines. Therefore, when the vehicle is in engine operation before running, only four carriages vibrate. The measurement result is consistent with this actual situation. The actual length of the four carriages is 86.64 m, and the DAS measurement result is 86.4 m, as shown in Figure 8a. There are two compressors at the head and tail of the vehicle, with only one compressor working at one time. When the vehicle stops running, the compressor on the train still needs to work for a period of time. The vibration caused by the compressor is expressed in Figure 8b; this result is also consistent with the actual situation.

Figure 9 shows the data of a full journey of the tested vehicle. The actual total length of the six carriages of the railway vehicle is 119.8 m, while the length measured by the DAS is 121.2 m and the spatial resolution of the DAS is 3 m, resulting in an error of ±3 m. The vehicle traveled a total length of 585.6 m in this operation.

As shown in Figure 10, the test of this full journey lasts approximately 40 s, and it can be divided into three sections: the acceleration section, the constant speed section, and the braking section. Acceleration, speed, and braking distance are important parameters that engineers need, and these three parameters can be calculated based on the measured data in Figure 10. The threshold extraction method in Figure 4c is used to find coordinates from Figure 10, including the starting and ending coordinates of the acceleration section. The coordinates contain the time and position information, and according to the formula Δs_1_ = a·Δt_1_^2^/2 (Δs_1_ and Δt_1_ are the distance interval and time interval from the starting point to the ending point of the acceleration section, respectively, and a is the acceleration), the value of the acceleration can be calculated. A similar method is used for the velocity calculation; the starting and ending coordinates of the uniform velocity section are extracted separately, and the speed can be calculated according to the formula Δs_2_ = v·Δt_2_ (Δs_2_ and Δt_2_ are the distance interval and time interval from the starting point to the ending point of the constant velocity section, respectively, and v is the constant velocity). The braking distance is obtained based on the starting and ending coordinates of the deceleration section. The acceleration, speed, and braking distance measured by the DAS are 1.376 m/s^2^, 78.98 km/h, and 198.4 m, respectively, while the results measured by the onboard software of the vehicle are 1.38 m/s^2^, 80 km/h, and 198.8 m, respectively.

Figure 11 shows the data of the railway vehicle running for 90 s; in this figure, the yellow data indicate the positive phase of vibration caused by the vehicle. It can be seen that there are seven clear yellow parallel lines that correspond to the locations of the axles from one to seven of the railway vehicle below.

## 4. Discussion

Most researchers who use distributed optical fiber sensors to achieve sensing generally lay the fiber cable parallel to the road. In this field test, there are two types of roads (refer to Figure 2) intersecting in two directions, the municipal road is perpendicular to the fiber cable, and the railway is parallel to the fiber cable. The data corresponding to the two kinds of roads were both analyzed. The novel aspect of this field test is that it provides the authors with an opportunity to observe some details that the pre-researchers did not notice, using the fiber cable perpendicular to the municipal road. 

For the municipal road test, the data located from 700 m to 800 m of the fiber cable were analyzed, which precisely correspond to the distribution of fiber cables on the municipal road. Figure 6 shows the results of six different kinds of vehicles, as well as the different weights of the vehicle results in different strain ranges on the fiber cable. The lighter the vehicle is, the smaller the strain range of the optical cable is; the heavier the vehicle is, the larger the strain range of the optical cable is. This phenomenon is similar to the driven signal strength on the PZT [18,19,20]. This is related to the vibration intensity caused by the weight of the vehicle body on the ground. When the vehicle body is heavy, the vibration is strong, and the strong vibration waves can propagate over a certain distance, causing the strain to cover a longer range of the optical fiber. Here, the interesting point is that the number of axles of different vehicles can be identified without using a camera, which can protect the privacy of the scene and vehicles. And also, in this test, because the direction of vehicle travel is perpendicular to the optical cable, when different types of vehicles pass through optical cables simultaneously, different positions of the optical cable will respond to the vibrations, so the vibration of parallel vehicles will not overlap.

Based on the data in Figure 7, the traffic flow, vehicle type, and vehicle speed can be calculated based on the length of the vehicle body and the time duration when the axles pass through the optical fiber. If a camera is used together with the DAS, the time interval Δt between the axles passing through the optical cable can be measured using the DAS. The vehicle type can be obtained from the camera and then the distance ΔL between axles can be obtained from the vehicle type database. Finally, the distance between the axles by the time interval (v = ΔL/Δt) and the running speed v of the vehicle can be calculated. For example, if the wheelbase of the motorcycle is 1.25 m (ΔL), the time interval (Δt) based on the data in Figure 6a is 0.5 s and then the speed of the motorcycle can be calculated, with it being about 7.2 km/h.

For the railway test, all the data along the fiber cable were analyzed, ranging from 0 m to 1000 m. The data in Figure 9 are the data of a full journey of the tested vehicle. The range of each full journey was not easy to measure using laser ranging technology, as the field test scene has curved sections. When engineers need to extract the length of a test journey, they need to manually pull a ruler on the railway each time; this is quite time consuming and greatly increases the workload of the engineers. Therefore, the DAS is a technology that can effectively solve this problem. And the parameters such as speed, acceleration, braking distance measured by the DAS in Figure 10 are very close to the results calculated by the onboard software. During the testing of newly manufactured vehicles, engineers often encounter software bugs; once these software bugs occur, they cannot continue the test, which significantly affects their work efficiency. Moreover, the software only provides average results, but the DAS data can provide more detailed parameters. If finer time intervals are used for the data in Figure 10, a more detailed acceleration distribution and velocity distribution can be obtained. The reliable DAS results can serve as a reference for engineers, even replacing the software. Also, the results of the DAS can play an important role in helping drivers to set safe acceleration values, braking distances, running speeds, etc. 

For the data in Figure 11, the vehicle has a total of 12 axles. The reason only seven axle lines can be seen is that, except for the first and last axles, for the axle lines from two to six, the distance between the two adjacent axles of the previous carriage and the rear carriage is very close at the connection of every two carriages in the middle, making it difficult to distinguish the two axles during vehicle operation. The spatial resolution of the DAS system is 3 m, which means that the event resolution is 6 m, and two adjacent vibrations can only be distinguished when the distance between two adjacent axles is greater than 6 m. However, for the vehicle under test, the distance between the two axles of two adjacent carriages is 4.65 m. Therefore, only seven axles were observed in the experiment because the two axles at the junction of the adjacent two carriages in the last 4.65 m cannot be distinguished by the 6 m event resolution. Furthermore, as the spatial resolution of the DAS unit used in the paper is 3 m, based on the acceleration of 1.38 m/s^2^, the speed of the vehicle in Figure 9 and Figure 10 exceeds 3 m/s after the third second, which means that the distance traveled by the vehicle in one second is greater than 3 m. Due to the limitation of the DAS’s spatial resolution, the axles in Figure 9 and Figure 10 cannot be identified. However, the maximum speed of the data in Figure 11 is only 2.45 m/s; thus, the resolution of the DAS is sufficient to support wheel identification in Figure 11.

All the above results prove that the data collected by the DAS contain many detailed parameters, which can calculate the various parameters that engineers need. While the huge amount of data is a critical problem which limits the implementation of DAS monitoring systems in the field [22]. The vehicle operation data features identified in this manuscript will help to develop real-time data processing methods using neural network [23] methods.

## 5. Conclusions

This paper develops the implementation of distributed acoustic sensors which can monitor municipal road transportation and railway transit. In municipal road flow monitoring, a DAS can monitor vehicle flow and speed and determine vehicle type based on experimental data. In railway transit, via analyzing the distribution of the sound waves in time and space, parameters such as the train’s length, running distance, acceleration, speed, and braking distance can be calculated. The measured results were compared with the results calculated by the onboard software of the railway vehicle, and the experimental results showed good consistency with the software-calculated results. The conclusion regarding the data characteristics obtained in this field test has important significance for developing the data processing method of DASs. All the results of the field test indicate that DASs have significant value in the safe operation of vehicles both for municipal roads and railways. DAS-based data can mine out many other important parameters through more detailed analyses, which require further exploration by researchers all over the world.

## Data Availability

The data that support the plots and maps within this paper and the other findings of this study are available from the corresponding author upon reasonable request.
